# Golgi Enrichment and Proteomic Analysis of Developing *Pinus radiata* Xylem by Free-Flow Electrophoresis

**DOI:** 10.1371/journal.pone.0084669

**Published:** 2013-12-26

**Authors:** Harriet T. Parsons, Cristina S. Weinberg, Lucy J. Macdonald, Paul D. Adams, Christopher J. Petzold, Timothy J. Strabala, Armin Wagner, Joshua L. Heazlewood

**Affiliations:** 1 Scion, Rotorua, New Zealand; 2 Joint BioEnergy Institute and Physical Biosciences Division, Lawrence Berkeley National Laboratory, Berkeley, California, United States of America; 3 Department of Bioengineering, University of California, Berkeley, California, United States of America; University of Hyderabad, India

## Abstract

Our understanding of the contribution of Golgi proteins to cell wall and wood formation in any woody plant species is limited. Currently, little Golgi proteomics data exists for wood-forming tissues. In this study, we attempted to address this issue by generating and analyzing Golgi-enriched membrane preparations from developing xylem of compression wood from the conifer *Pinus radiata*. Developing xylem samples from 3-year-old pine trees were harvested for this purpose at a time of active growth and subjected to a combination of density centrifugation followed by free flow electrophoresis, a surface charge separation technique used in the enrichment of Golgi membranes. This combination of techniques was successful in achieving an approximately 200-fold increase in the activity of the Golgi marker galactan synthase and represents a significant improvement for proteomic analyses of the Golgi from conifers. A total of thirty known Golgi proteins were identified by mass spectrometry including glycosyltransferases from gene families involved in glucomannan and glucuronoxylan biosynthesis. The free flow electrophoresis fractions of enriched Golgi were highly abundant in structural proteins (actin and tubulin) indicating a role for the cytoskeleton during compression wood formation. The mass spectrometry proteomics data associated with this study have been deposited to the ProteomeXchange with identifier PXD000557.

## Introduction

Understanding secondary cell wall formation in coniferous species has long been a research priority of the forestry industry with respect to timber and pulp production [1]. With the more recent utilization of this feedstock as a source of high-value woody biomass for biofuels this resource is of even more importance than ever [2]. The principal species employed by global forest industries comprise members from the *Pinus* spp. and *Eucalyptus* spp. [3]. The main polysaccharide components of developing xylem cell wall in coniferous species such as pine are galactoglucomannans, heteroxylans and cellulose, with some arabinogalactan proteins [4,5]. The distribution of polysaccharides varies considerably within one tree. One such example is compression wood, which is a type of reaction wood found in conifers that enables bent trees to return to the vertical position [6] and can contain more than 10% w/w of β-(1,4)-galactan [6–8] a polysaccharide that in coniferous species is found almost exclusively in compression wood [6].

High levels of galactan reflect the severity of the compression wood present in pine [6]. The physiological role of β-(1,4)-galactan is currently not well understood, but it has been proposed to strengthen the secondary cell wall, absorb mechanical stresses and to aid in the generation of compression forces [9]. Recent experimental evidence shows that β-(1,4)-galactan is correlated with post-harvest/post-processing wood instability, particularly longitudinal shrinkage [10–12]. Compression wood can be found throughout the stems of fast-growing *P. radiata* trees in commercial plantation forests [10]. Decreasing or modulating the levels of β-(1,4)-galactan in such trees is expected to improve wood quality in pine species such as *P. radiata* by improving dimensional stability. Galactan synthase (GalS) is a key enzyme in compression wood formation as it catalyzes the transfer of UDP-galactose on to the growing β-(1,4)-galactan [6,13]. An assay for its activity has been developed and high levels of activity have been detected in microsomal fractions [6] suggesting that it is either Golgi-localized, or possibly in transit to the apoplast/plasma membrane.

Understanding the changes that accompany different types of wood formation would clearly be of great benefit to the forestry industry. A recent study outlines transcriptomics changes associated with compression wood formation [14] but almost nothing is known about the protein content of woody species and even less about how this relates to compression wood formation. Targeting Golgi-localized enzymes involved in the production of non-cellulosic and pectic polysaccharides could alter cell wall properties and potentially improve timber characteristics and saccharification efficiency [15], thereby increasing efficiency in both the timber and biofuel industries. 

Several attempts at characterizing proteins associated with cell wall biosynthesis have been made but very few Golgi proteins were identified. Initial proteomics studies into coniferous gymnosperm cell wall biosynthesis focused on soluble proteins [16,17]. A more recent proteomics approach focused on membrane-associated proteins and used detergent–based phase separation as a Golgi membrane protein enrichment technique [13]. Unfortunately, protein identification was limited by poor sample recovery because of additional purification steps needed to overcome protein-cytoskeleton interaction. Of the 175 proteins identified in a previous analysis of membrane proteins, only two are known to localize to the Golgi [13], none of which were good candidates for GalS, despite substantial purification of this activity in the procedure. A number of polysaccharide biosynthesis proteins were identified at low levels amongst a complex background of integral membrane proteins from contaminating organelles [13], suggesting that whilst such an approach was feasible, further improvements in purification techniques were required. 

In a recent study aimed at expanding the Golgi proteome of the model dicotyledonous, herbaceous species Arabidopsis, a new technical approach was adopted which permitted Golgi membranes to be isolated to around 80% purity [18]. Using a combination of Free Flow Electrophoresis (FFE) and mass spectrometry resulted in 371 Golgi proteins being identified from Arabidopsis cell suspension-cultures [18]. In the current study, this approach was applied to both severe and mild compression wood from *P. radiate*. Developing xylem was chosen as the starting tissue as these cells are producing relatively thick secondary cell walls. Although the identification of Golgi-localized enzymes involved in secondary cell wall production in a coniferous species was a key aim in this study, these contrasting tissue types were also selected in attempt to identify galactan synthase candidates. This report is the first demonstration of the utility of FFE in increasing the purity of organelle isolates from a non-model woody species. It achieves significant increases in both the purity of Golgi preparations and the number of identified Golgi-localized proteins, as compared to previous studies. This will serve as a platform for targeting Golgi-localized cell wall biosynthetic pathways for the wood and biofuel production industries in the future.

## Materials and Methods

### Ethics Statement

Permission to harvest plant material was obtained from Steve R. Auten, Resource Manager for the Swanton Pacific Ranch (California Polytechnic State University, San Luis Obispo, USA). Material was selected from remaining trees from a recently harvested pine plantation and did constitute protected land or environmentally sensitive material.

### Preparation of Golgi-enriched developing xylem fractions

A three-year-old pine tree (*Pinus radiata* D. Don) was sourced from Swanton Pacific Ranch, Davenport, CA, USA (California Polytechnic State University, San Luis Obispo) in June 2011. Leaning trees were identified from a pine plantation to provide a sources of compression wood (Figure S1 in File S1). The medium part of a selected tree was felled and cut into 40cm sections (logs) which were immediately placed on ice. Logs were stripped of bark within 3 h of felling and the developing xylem on the exposed stem was carefully stripped away of the log sections that contained developing severe compression wood (sCW) and mild compression wood (mCW) sourced from the opposite side of the harvested logs. Wood disks from the same stem sections were saved for chemical analysis. Strips of developing xylem (ca. 40g) from opposing sides of several logs corresponding to sCW and mCW were quickly chopped into 2 mm sections using razor blades in ice-cold homogenization buffer containing cOmplete Protease Inhibitor, EDTA-free (Roche, USA), 10 mM Na_2_HPO_4_ pH 7.2, 1% Dextran 200,000, 0.4 M sucrose, 3 mM EDTA and 2 mM DTT) in a 1:1.2 (w/v) ratio. Extraction of organelles from the xylem homogenate was maximized by gentle agitation of the homogenate at 4°C for 45 min prior to filtration through cheesecloth. The filtered homogenate was centrifuged at 5000 × *g* for 10 min. The supernatant was then carefully layered onto a sucrose cushion (1.6 M sucrose in homogenization buffer) and centrifuged at 100,000 × *g* for 90 min. The supernatant was replaced with a step gradient of 1.0 M sucrose (10 ml), 0.75 M sucrose (10 ml) and 0.2 M sucrose (4 ml), taking care not to disturb the cushion of cellular contents. The gradient was then centrifuged at 100,000 x *g* for 90 min. All sucrose gradient buffers were made up in homogenization buffer. Golgi membranes were collected from the 0.75/1.0 M sucrose interface and stored overnight at 4°C. 

### Free flow electrophoresis

**Figure 1:**
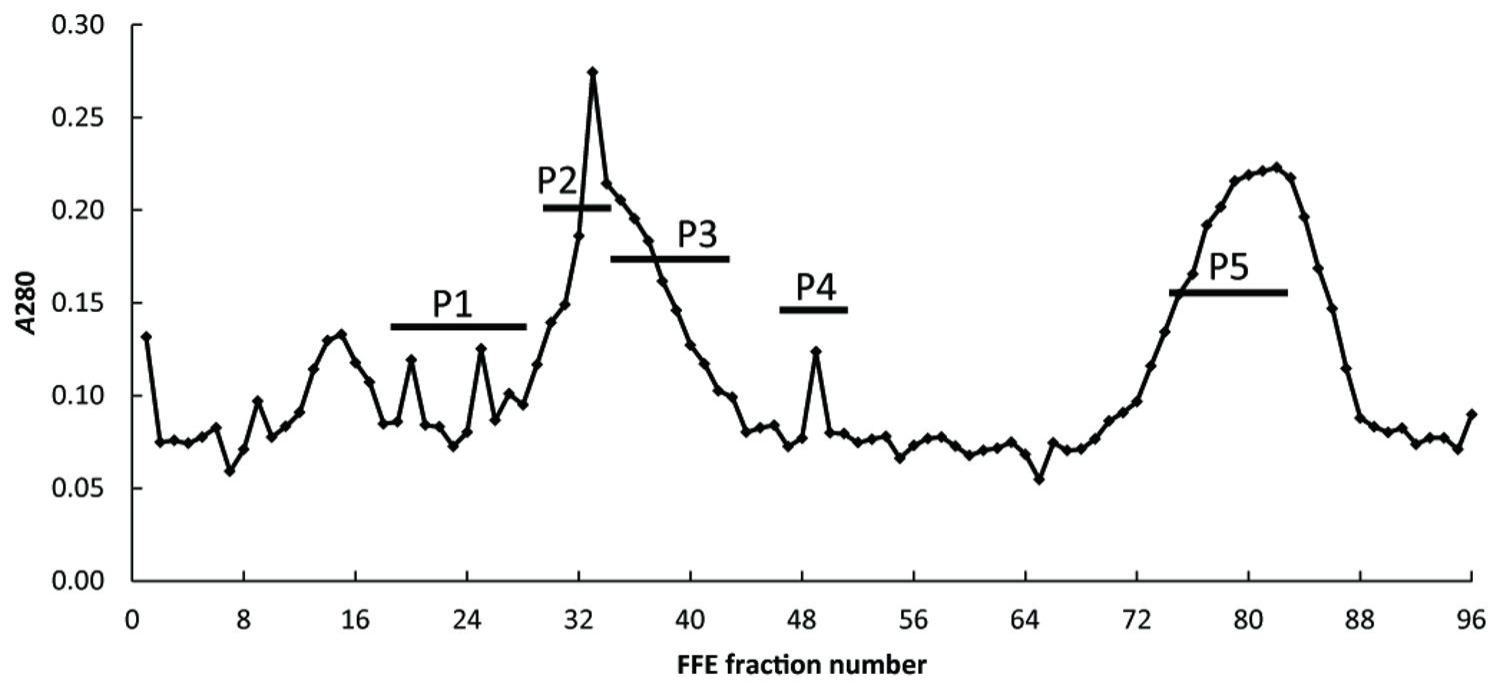
Post-FFE protein profile of enriched Golgi membrane preparations from strong compression wood (sCW) of developing pine xylem. FFE separated samples are distributed into 96-wellplates and protein distribution can be monitored by absorbance (A_280_ nm). The post-FFE profile for both the mCW and sCW samples derived from the 0.75/1.0 M interface had similar profiles. The FFE fractions were pooled as follows: P1; fractions 16 to 30, P2; fractions 31 to 33, P3; fractions 34 to 40, P4; fractions 48 to 50 and P5; fractions 72 to 80. CW-P1 to P5 were concentrated and analyzed for GalS activity (Table S5 in File S1).

Free-flow electrophoresis (FFE) was performed using a Free Flow Electrophoresis System (FFE Service, Germany) with a separation chamber height of 0.5mm at 8°C. A detailed explanation of the operation and use of the instrument to separate the plant endomembrane has been recently outlined [19]. In summary, FFE buffers were prepared as follows: Separation buffer: 280 mM sucrose, 10 mM acetic acid, 10 mM triethanolamine, 1 mM EDTA, pH to 7.0 with NaOH; Stabilization buffer: 200 mM sucrose, 100 mM acetic acid, 100 mM triethanolamine, 10 mM EDTA, pH to 6.5 with NaOH; Anodic and cathodic electrode buffers: 100 mM acetic acid, 100 mM triethanolamine, 10 mM EDTA, pH to 6.5 with NaOH. A voltage of 680 to 700 V was applied to the membrane samples, resulting in a current of 132 to 137 mA. Media flow rate was set to 250 ml h^-1^, sample flow rate to 2500 µL h^-1^. Fractions were collected on pre-cooled 2-mL 96-well plates, and then pooled according to the peaks of interest identified after detection (A_280_ nm) into five fractions (Figure 1). Membranes were collected from fractions by centrifugation at 50,000 × *g* for 45 min, re-suspended in ice cold 10mM Tris-HCl, pH 7.5, and stored at -80°C. 

### GalS assay

Standard GalS assays were performed according to [6]. Protein quantitation was performed using the Bradford assay (Bio-Rad, USA) according to the manufacturer’s instructions. Protein (10ug) was combined with 25µM 2AP-Gal_7_ or Gal_9_, 1.5mM UDP-Gal, 40mM MES-KOH (pH 6.5), 0.75% Triton X-100, 25mM MnCl_2_, 20mM NaF and 160mM sucrose. Reactions were incubated for 2 h at 25°C. Reactions were terminated by heating samples for 5 min at 95°C. Samples were diluted with 150µl water, and insoluble material was removed by centrifugation at 14,000 × g for 5 min. The supernatant was dried in a SpeedVac concentrator, resuspended in 70% acetonitrile, re-centrifuged and the supernatant separated by normal-phase HPLC. HPLC was conducted on an Agilent 1290 HPLC machine using a cosmosil/cosmogel D (Nacalai Tesque Inc., Japan) of (4.6mm x 250mm). Separation of compounds was monitored by fluorescence detection. The column was equilibrated in 70% solvent B (solvent A: ammonium formate solution of pH 4.1, Solvent B: 100% acetonitrile) and eluted with the following gradient from 70%B to 50%B over 30 minutes at a flow rate of 1mL min^-1^ at 40°C. Activity was recorded as pmol Gal min^-1^mg^-1^protein.

### Liquid chromatography tandem mass spectrometry (LC-MS/MS)

Fractions collected from FFE separation of microsomal samples were digested with trypsin (1:10 w/w) overnight at 37 °C in 40% methanol and 10mM Tris-HCl (pH 7.8). After overnight digest, samples were dried using a SpeedVac concentrator. Prior to mass spectrometry, peptide samples were rehydrated in a solution of 2% acetonitrile (v/v) and 0.1% formic acid (v/v). Samples (1 µg) were analyzed using a TripleTOF™ 5600 System (AB SCIEX) with an Eksigent NanoLC™-1D System (AB SCIEX) incorporating a PepMap100 C18 μ-Precolumn and a 75 µm × 15 cm PepMap™ C18 reverse-phase column (Dionex) at a flow rate of 300 nL/min. Peptides were eluted over a 60 min gradient (2% to 80% acetonitrile) using the buffers 2% acetonitrile (v/v) with 0.1% formic acid (v/v) and 98% acetonitrile (v/v) with 0.1% formic acid (v/v). For each cycle, a maximum of 30 precursor ions between 400 and 1250 m/z, with charges from 2 to 5, a minimum threshold of 150 cps, using Q1 Unit Resolution, were automatically selected for collision-induced dissociation (CID) using Rolling Collision Energy for 50 ms by the Information Dependent Acquisition (IDA) capabilities of Analyst TF v1.5.1 (Build 2756). Product ion data from CID were collected between 100 and 1600 m/z. Precursor ions and their isotopes were excluded from further selection for 16 sec with a mass tolerance of 0.05 Da.

### Tandem mass spectrometry data analysis

Microsomal fractions separated by FFE that contained high GalS activity from samples mCW and sCW(fractions P2 and P3) were each subjected to in-depth characterization by LC-MS/MS. Raw data were exported as .mgf files using the MS Data Converter tool (v1.1 Beta) available through the PeakView™ Software v1.1.1.2 (AB SCIEX) using default settings. These data files were interrogated with the Mascot search engine version 2.3.02 (Matrix Science, UK) with a peptide tolerance of ±50 ppm and MS/MS tolerance of ±0.1 Da; variable modification was Oxidation (M); up to one missed cleavage for trypsin; Group protein families; and the instrument type was set to ESI-QUAD-TOF. Searches were performed against a Viridiplantae protein database created from GenBank [20] using RefSeq [21] as of May 2012 (521619 sequences and 199111130 residues) or all available nucleotide sequences for *Pinus* spp. filtered using the taxonomy option at GenBank as of July 2012 (1477602 sequences and 184443200 residues) or the *Arabidopsis thaliana* protein set (TAIR10) available from The Arabidopsis Information Resource (35393 sequences and 14486052 residues). A false discovery rate and ions score or expected cut-off was calculated for each fraction using the Decoy feature of Mascot on the MS/MS Ions Search interface. A significance threshold corresponding to a false discovery rate of ≤ 5% (*p*<0.05) was used to determine the ‘Ions score or expect cut-off’ for peptide matches. An ‘Ions score or expect cut-off’ value for each data file against database was applied. Ions score or expect cut-off for databases were the following (mCW = mild compression wood and sCW = severe compression wood), Arabidopsis: 29 (mCW-P2), 29 (mCW-P3), 29 (sCW-P2), 29 (sCW-P3); Viridiplantae: 41 (mCW-P2), 41 (mCW-P3), 40 (sCW-P2), 40 (sCW-P3); *Pinus* spp: 40 (mCW-P2), 40 (mCW-P3), 39 (sCW-P2), 39 (sCW-P3). Ions score is −10log(P), where P is the probability that the observed match is a random event. Ions scores ≥ the cut-off value indicate identity or extensive homology (*p*<0.05). Specific false discovery rates for peptide matches above the identity threshold were the following, Arabidopsis: 2.09% (mCW-P2), 1.34% (mCW-P3), 1.46% (sCW-P2), 1.46% (sCW-P3); Viridiplantae: 2.09% (mCW-P2), 1.79% (mCW-P3), 1.74% (sCW-P2), 1.37% (sCW-P3); *Pinus* spp: 2.56% (mCW-P2), 0.6% (mCW-P3), 1.16% (sCW-P2) 1.61% (sCW-P3).

### Tandem mass spectrometry data availability

The mass spectrometry proteomics data have been deposited to the ProteomeXchange Consortium (http://proteomecentral.proteomexchange.org) via the PRIDE partner repository [22] with the dataset identifier PXD000557 and DOI 10.6019/PXD000557. The data comprises the raw data files (.wiff AB Sciex), the peak files (.mgf) and the search files for interrogation against Arabidopsis (Mascot .dat) for samples mCW-P2 (31966), mCW-P3 (31967), sCW-P2 (31968) and sCW-P3 (31969).

### Chemical analysis of wood samples

The neutral sugar content of wood samples was determined according to a previously described procedure [23]. A total of three wood samples harvested from either the mCW and sCW tissue were analyzed from near the region where developing xylem were harvested.

## Results

### Enrichment of Golgi-derived membranes from developing pine xylem

Membrane enrichments were prepared from developing pine xylem from severe compression wood (sCW) and mild compression wood (mCW) from a single pine tree. Developing xylem from opposing sides of the trunk were chosen in an attempt to increase the likelihood of identifying a valid GalS candidate in the sCW samples. Neutral sugar analysis of alcohol insoluble fractions from these samples showed a significant increase in galactose content; indicating a strong induction of compression wood in the sCW samples (Table 1). Membrane enrichments employed a modified version of previously outlined methods to enrich Golgi membranes [13,18]. These approaches ensured Golgi stack integrity was preserved as much as possible during the initial enrichment procedure. Free flow electrophoresis was then performed on membrane enrichments as previously applied in the model plant Arabidopsis [18]. The presence and enrichment of Golgi membranes throughout the procedure was monitored by assaying for galactan synthase (GalS) activity. Compression wood is characterized by high levels of β-1,4-galactan which is generally thought to be synthesized by Golgi-localized galactan-synthesizing enzymes [6–8]. GalS activity was relatively low in the developing xylem homogenate in both mCW and sCW samples (Table 2). Golgi membranes were enriched by density centrifugation of the xylem homogenate. Most GalS activity was expected to be found in the same fractions on the gradient as that in which Golgi membranes are typically found (0.75/1.0 M interface). This was indeed the case for the membrane enriched mCW samples, but for the sCW samples GalS activity was higher in the 1.0/1.6 M sucrose interface fractions (Table 2). The 1.0/1.6 M interface represents membranes and organelles which are too dense to migrate through 1.0 M sucrose at 100,000 × *g*. Fewer Golgi membranes may have floated up to the 0.75/1.0 M interface in the sCW samples if they were more closely associated with other organelles and membranes when compared to mCW. The GalS activity could not be measured until after membranes had been loaded for FFE. As the 1.0/1.6 M interface was expected to contain contaminants and a clear band of membranes was present at the 0.75/1.0M sucrose interface for the sCW sample, FFE was therefore still performed using membranes from the 0.75/1.0M interface.

**Table 1:** Neutral sugar analysis (mM) of mild and severe compression wood samples.

**Sample**	**Arabinose**	**Galactose**	**Glucose**	**Xylose**
mCW	0.39 ± 0.00	0.39 ± 0.00	8.39 ± 0.00	2.97 ± 0.00
sCW	0.34 ± 0.02	1.20 ± 0.02	7.37 ± 0.03	2.57 ± 0.03

A total of three wood samples from the regions used to harvest developing xylem were analyzed (*n*=3).

**Table 2:** Activity of the GalS marker enzyme in pine homogenates and sucrose gradient interfaces.

**Sample**	**Specific activity**	**Total activity**
	**[pmol min^-1^ mg^-1^protein]**	**[pmol min^-1^]**
mCW Homogenate	183.2	7336.4
mCW Sucrose interface (0.75/1.0 M)	430.5	3101.0
mCW Sucrose interface (1.0/1.6 M)	393.8	1301.7
mCW-P2	7304.9	29.2
mCW-P3	753.9	67.8
sCW Homogenate	182.0	9715.4
sCW Sucrose interface (0.75/1.0 M)	622.6	3231.2
sCW Sucrose interface (1.0/1.6 M)	1105.7	3811.5
sCW-P2	12318.8	197.1
sCW-P3	34662.8	588.0

### Increasing the purity of Golgi membranes using FFE

Golgi cisternae from Arabidopsis cells have previously been found to migrate to the anodic edge of a main protein peak consisting of ER, mitochondria and peroxisomes [18]. Based on these observations and on the protein spectrum at A_280_ nm after FFE separation of pine membrane samples, FFE fractions were pooled as follows: P1; fractions 16 to 30, P2; fractions 31 to 33, P3; fractions 34 to 40, P4; fractions 48 to 50 and P5; fractions 72 to 80 (Figure 1). Generally, the highest specific GalS activity was found in fractions P2 and P3 for both mCW and sCW samples (Table 2). Comparatively, at least 10-fold lower GalS activity levels were measured in fractions P1, P4 and P5 (Table S5 in File S1) for both mCW and sCW, except for fraction P1 from the mCW sample which had similar activities to fraction P3. As anticipated, GalS activity was higher in sCW than mCW fractions. The activity of P2 was about 2.8-fold lower than that of P3 but in mCW fractions the highest activity was found in P2. The lower levels of GalS activity and low protein yields in fractions P1, P2 and P5 prompted further characterizations by mass spectrometry to be confined to fractions P2 and P3 for both mCW and sCW.

### Characterization of post FFE fractions by mass spectrometry

A fully annotated genome is not yet available for any pine species which can create some difficulty when attempting to identify proteins by mass spectrometry. As the number of publicly available pine sequences is limited, and in an attempt to identify as many proteins as possible, we assembled three separate in-house sequence databases. The current Arabidopsis protein set (TAIR10), all Viridiplantae proteins (GenBank) and all *Pinus* spp. nucleotide sequences (GenBank). The four samples comprising mCW-P2, mCW-P3, sCW-P2 and sCW-P3 were each analyzed against the three databases using the Mascot search engine. Data for all peptide and protein matches are outlined in Tables S1 (Arabidopsis), Table S2 (Viridiplantae) and Table S3 (*Pinus* spp.) in File S1. Generally, these searches yielded a comparable number of protein identifications for each fraction, although on average searches against both pine nucleotides and Viridiplantae proteins resulted in ~300 proteins per fraction while searches against Arabidopsis resulted in ~200 proteins per fraction (Tables S1-S3 in File S1). The Viridiplantae database resulted in the largest number of unique proteins (845 identified), followed by the pine database (615 identified) and Arabidopsis with 397 identified proteins (Table 3).

**Table 3:** Summary of search results after LC-MS/MS analysis of post-FFE fractions mCW-P2, mCW-P3, sCW-P2 and sCW-P3 using the Mascot search engine.

**Proteins**	**Arabidopsis**	**Viridiplantae**	***Pinus* spp.**
Total Unique^1^	397	845	615
Total Grouped	262	349	310
Total endomembrane (grouped)	81	108	119
Total Golgi (grouped)	15	19	21

^1^ Total unique numbers represent unique proteins (by identifier) after combining search results from all fractions after each database search result.

Proteins identified from the four samples mCW-P2, mCW-P3, sCW-P2 and sCW-P3 were manually assigned a functional and subcellular classification. Functional assignments were made using annotations directly from a proteins description or after using BLAST interrogations of annotated protein datasets such as those available for UniProtKB/Swiss-Prot or Arabidopsis. Proteins of sufficient homology to Arabidopsis could be assigned a subcellular localization from the SUBcellular Arabidopsis (SUBA) database [24]. This enabled the creation of a data matrix for proteins identified from the different databases and fractions analyzed by LC-MS/MS (Table S4 in File S1). This functional grouping of identified proteins revealed that although the total number of protein types identified was significantly greater when either the Viridiplantae and pine databases was used, the total number of distinct functional proteins was similar to numbers when using the Arabidopsis database (Table 3). This indicates that the number of redundant entries or similar entry types in the Viridiplantae and pine databases is relatively large. This is not too surprising given the former comprises proteins from a diverse range of species and the latter is a highly redundant dataset. The total number of endomembrane proteins (ER, Golgi apparatus, trafficking, vascular, plasma membrane and extracellular) comprised around a third of the protein types identified, with Golgi proteins comprising about 20% of this group (Table 3). This demonstrates that while there are clear advantages to using incomplete species-specific datasets, in this instance nearly 1.5 million pine nucleotide sequences, a comparable number of protein identifications can be obtained from matching cross-species to multiple plant backgrounds (over 500,000 sequences for Viridiplantae). Most surprising was the number significant matches to the Arabidopsis database. While only comprising around 27,400 distinct loci, the total number of matches and overall identified Golgi proteins was similar after filtering redundant matches from the other identified datasets.

### Assessment of Golgi separation by FFE

The success of FFE in separating Golgi membranes from contaminants in developing pine xylem was assessed by following the migration of proteins for which a subcellular localization could be assigned. The P2 fractions represented the anodic edge of the main protein peak, where Golgi membranes were expected to migrate [18], and P3 fractions the cathodic edge. In an attempt to assess the performance of membrane separations, we employed a normalized protein count for all proteins functionally allocated in Table S4 in File S1. In the P2 fraction, an increase in the number of Golgi-localized proteins identified in the Arabidopsis, Viridiplantae or pine datasets was observed, indicating that some separation of Golgi membranes from contaminating membranes and organelles was occurring during electrophoresis (Table 4). There was little apparent difference in total Golgi identifications between the mCW and sCW samples, although minor increases were observed for ER and endomembrane proteins in the sCW samples. In the mCW sample, a small decrease in ER and trafficking proteins was also apparent in P2 compared to P3 but this was not apparent in the sCW sample (Table 4). Golgi membranes would be expected to contain a higher proportion of proteins involved in cell wall biosynthesis. Correspondingly, there seemed to be an increased number of proteins involved in cell wall biosynthesis in mCW-P2 than mCW-P3. Although again, little difference between fractions sCW-P3 and sCW-P2 (data not shown), supporting the idea of biochemical composition of samples affecting organelle separation. No apparent differences were observed between protein numbers for most of other subcellular compartments, although decreases in the total number of mitochondrial protein identifications was observed in the P3 fractions for both mCW and sCW samples (Table 4).

**Table 4:** Normalized protein counts highlighting changes in subcellular localization composition in samples mCW and sCW.

	**mCW-P2**		**mCW-P3**		**sCW-P2**		**sCW-P3**		**mCW**	**sCW**
	Proteins	% total	Proteins	% total	Proteins	% total	Proteins	% total	change	change
Golgi	84	8.6	66	7.4	59	8.2	48	7.1	+	
trafficking	85	8.7	106	11.9	68	9.4	64	9.5		
ER	37	3.8	48	5.4	46	6.4	39	5.8		+
endomembrane	66	6.7	70	7.9	69	9.6	76	11.3		+
vacuole	3	0.3	9	1.0	5	0.7	1	0.1		
PM	55	5.6	64	7.2	48	6.7	46	6.8		
extracellular	10	1.0	10	1.1	8	1.1	0	0		
structural	19	1.9	22	2.5	13	1.8	14	2.1		
cytosol	154	15.7	128	14.4	90	12.5	94	14.0		
mitochondria	142	14.5	82	9.2	79	11.0	66	9.8	+	
plastid	36	3.7	30	3.4	26	3.6	26	3.9		
peroxisome	21	2.1	13	1.5	12	1.7	15	2.2		
nucleus	11	1.1	9	1.0	11	1.5	11	1.6		+
translation	115	11.7	79	8.9	113	15.7	106	15.8		++
miscellaneous	142	14.5	154	17.7	73	10.1	67	10.0	++	
**Total identifications**	**980**		**890**		**720**		**673**			

Number of protein represent the total count of all proteins (a unique identifier) allocated to each subcellular location for each fraction as outlined in Table S4 in File S1.In the previous study on pine membrane proteins from developing compression wood, cytoskeletal proteins were found to be the most abundant subcellular category of proteins [13]. Cytoskeletal proteins (actins and tubulin) were still the most abundant proteins in both the mCW and sCW samples based on matched spectra (Tables S1-S3 in File S1). Slightly fewer cytoskeletal proteins were identified in the P2 fractions than the P3 fractions in both the mCW and sCW samples (Table 4), indicating that FFE may have some impact in separating membranes from cytoskeletal proteins. Overall more than 30 Golgi-localized protein functional classes were characterized from FFE separated Golgi membranes of pine (Table 5). Only two of these proteins had been previously characterized from membranes isolated from developing pine compression wood [13], namely UDP-xylosyltransferase, XXT and cellulose synthase-like A, CSLA (Table 5). Overall, FFE contributed to the purity of Golgi membranes, especially in mCW, but refinements to methods, both at the initial enrichment stage and the FFE stage, would be necessary before an equivalent degree of purification to Arabidopsis could be achieved [18].

**Table 5:** Golgi localized proteins identified from samples mCW and sCW harvested from developing xylem of pine.

**Golgi apparatus**	**mCW-P2**	**mCW-P3**	**sCW-P2**	**sCW-P3**	**Mast et al.**, [13]
cellulose synthase-like A, CSLA (GT2)	X	X	X	X	X
galacturonosyltransferase 8, GAUT8 (GT8)	X	X	X	X	
galacturonosyltransferase 9, GAUT9 (GT8)		X	X		
GAUT14 (GT8) like protein			X		
Galactosyltransferase family protein (GT31)	X				
UDP-xylosyltransferase, XXT (GT34)	X	X	X	X	X
GUT1 exostosin family (GT47)	X	X	X	X	
Exostosin family (GT47)		X	X		
TMN9/endomembrane 70	X	X	X	X	
nucleotide-diphospho-sugar transferase superfamily	X	X		X	
nucleotide/sugar transporter family protein			X	X	
protein of unknown function (DUF288)	X	X	X	X	
S-adenosyl-L-methionine-dependent methyltransferase	X	X	X	X	
Trichome Birefringence-Like, TBL (DUF231)	X	X	X	X	
UDP-D-glucuronate 4-epimerase (GAE)	X	X	X	X	
UDP-xylose synthase (UXS)	X	X	X	X	
UDP-arabinose 4-epimerase (UXE)	X				
reversible glycosylated polypeptide (RGP)		X			
SNARE family	X	X	X	X	
alpha/beta-hydrolases superfamily protein	X	X	X	X	
beta-1,2-N-acetylglucosaminyltransferase (CGL/GNT)	X		X		
alpha-mannosidase 1	X				
O-fucosyltransferase family protein	X	X	X		
O-glycosyl hydrolase (GH47)		X			
Copper transporting ATPase		X			
CASP CCAAT-displacement protein	X	X	X		
cation-chloride co-transporter	X	X			
cation efflux family protein	X		X		
calcium ion binding			X		

### Functional Analysis of sCW vs. mCW FFE fractions by LC-MS/MS

**Figure 2:**
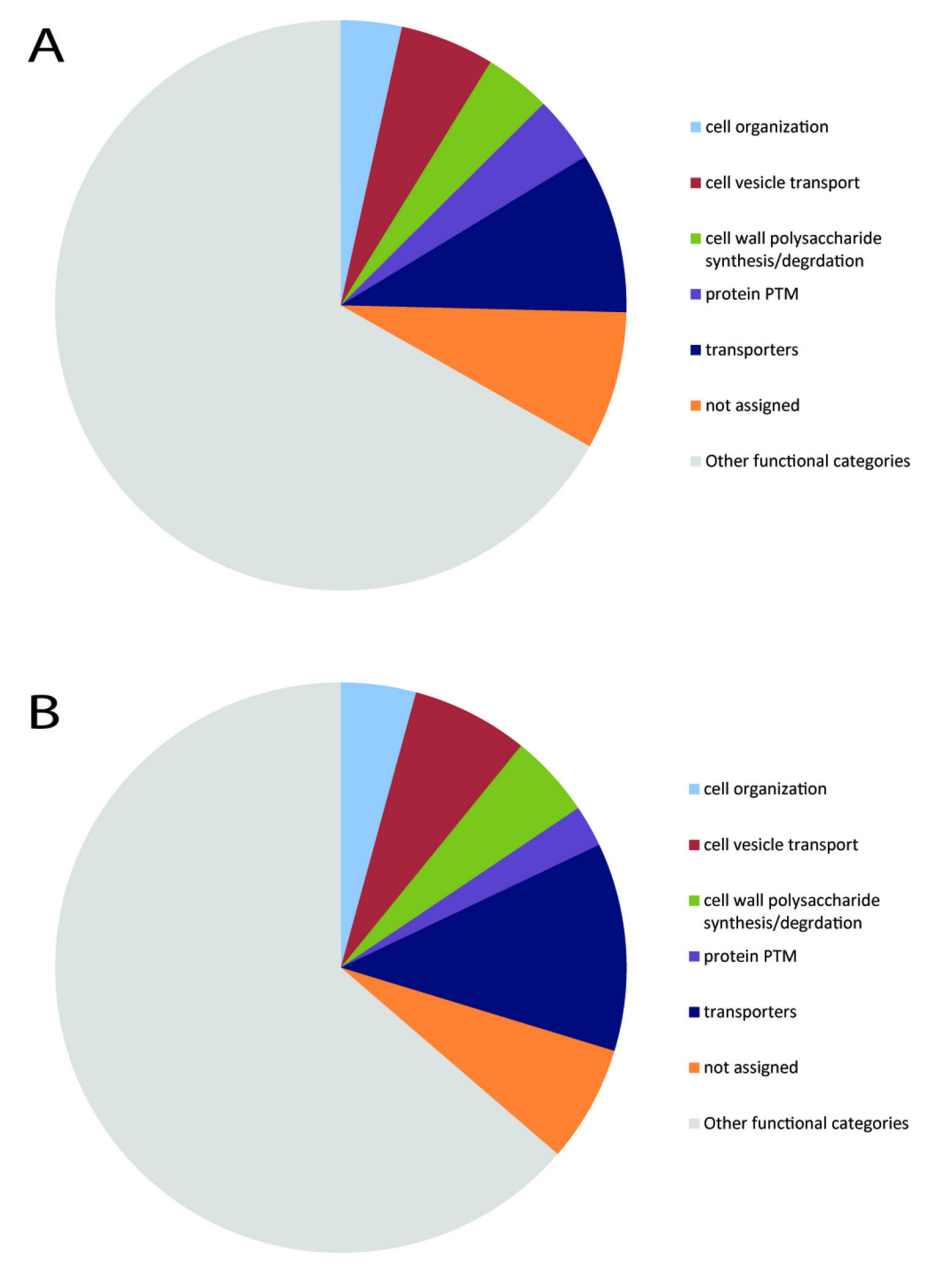
Differences in MapMan functional categories for proteins identified from mCW and sCW samples. Proteins identified from mCW (**A**) and sCW (**B**) samples using the Arabidopsis database were allocated into MapMan bins [25]. Only functional categories with differences are shown. Functional classes with little discernable difference were allocated to ‘other functional categories’.

With the aim of revealing broad differences in metabolism and subcellular processes between mild and severe compression wood, proteins that could be identified from the Arabidopsis database were assigned to MapMan [25] functional categories (Table S6 in File S1). No differences were apparent in cell wall synthesis, cell organization, protein post-translational modification or vesicle trafficking categories (Figure 2), though an increased number of plasma membrane and endomembrane-localized transporters resulted in an increase in the ‘transporter’ category in sCW (Figure 2). Functional categorization may, however, mask important differences at the individual protein level, especially as it is only applicable to proteins identified in the Arabidopsis sequence database.

Golgi-localized proteins involved in cell wall synthesis show some differences and similarities between sCW and mCW. In both, family GT8 is well represented with three members in sCW and two in mCW, when combining the total number of protein identifications from Arabidopsis, Viridiplantae and the pine dataset. There are two members of GT47 present in each, one of which shows homology to Arabidopsis GUT1 (Table 5). Many proteins with homology to the CSLA clade of GT2 were identified in both sCW and mCW, as were SAM dependent methyltransferases. UDP-xylose synthase (UXS) homologues were also quite frequently identified in both (Table 5).

The qualitative nature of this study does not permit for precise comparisons to be drawn between the sCW and mCW samples, although broad evaluations can be made. However, comparisons are further complicated by more proteins being identified in mCW than sCW (Table 4). Therefore it is difficult to comment on proteins identified only once. RGP1, however, was identified multiple times in mCW but never in sCW. A protein described as ‘GT8 like’ in GenBank [20] and a nucleotide sugar transporter were unique to sCW. TMN9/endomembrane 70 proteins were identified frequently but appeared more frequently in the mCW sample compared to the sCW sample. 

Proteins involved in clathrin mediated endocytosis and ER-Golgi trafficking were identified several times in sCW and mCW. Two classes of trafficking proteins, syntaxin of plants (SYP) family proteins and vacuolar sorting receptor proteins (VSR) were identified multiple times in mCW fractions but were absent in sCW, indicating a shift in post-Golgi trafficking pathways in sCW compared to mCW (Table S1 in File S1). SNARE family proteins were identified less often in sCW whilst NSF attachment proteins were absent. This corroborates the idea of a shift in post-Golgi trafficking pathways in cells synthesizing compression wood, although a more targeted study would be required before any conclusions could be made.

Taken together, this suggests that cell wall metabolism in both the Golgi and trafficking of cell wall components was altered in sCW compared to mCW. An actin depolymerizing factor was identified in both mCWP2 and mCWP3 but was absent from sCW, suggesting changes in cell structure in compression wood developing xylem. The increase in membrane purity after FFE enabled better coverage of the pine secretory system than was previously possible, allowing such differences to be observed.

## Discussion

Many proteins involved in the biosynthesis of non-cellulosic and pectic polysaccharides are known to be localized in the Golgi apparatus [18,26–28]. Cell wall biosynthesis is of critical importance in timber-producing species such as pine, which has the potential to become a source of biofuels in the future. From a commercial perspective, an increased understanding of how different wood types are synthesized would help production of trees with more homogenous timber. Avoiding excess compression wood formation would also be an advantage as the high lignin content and structural properties of compression wood are a disadvantage in the pulp and paper industries. Non-cellulosic polysaccharides such as xylan and glucomannan are thought to play key roles in lignin deposition in compression wood [5] whilst β-1,4-galactan is one of the main components in compression wood [4]. Therefore a Golgi proteome for these species would be a major asset in guiding targeted manipulation of cell wall properties. However, Golgi stacks are unstable during subcellular fractionation [18,29] and they represent only a low proportion of the total membrane body within the cell [30]. Consequently, previous attempts at proteomics analyses of the Golgi in pine species have yielded very limited information. This study preferentially applied FFE to the isolation of Golgi membranes from a woody species and consequently marks a significant improvement on the number of known Golgi-localized proteins identified within isolates. Previously, an attempt to purify Golgi from *P. radiata* [13] identified only two known Golgi-localized proteins amongst high background levels of contaminants from other membrane systems and cytoskeletal proteins. Although considerable contamination from other membrane systems was evident in our samples, the FFE was able to provide an enriched fraction that provided fraction with 5 to 10 % Golgi proteins., Thus in this study, using a combination of sequence databases, 30 distinct protein functional types could be confidently assigned as being derived from the pine Golgi apparatus (Table 5). 

### Free-flow electrophoresis and the enrichment of Golgi membranes

Using GalS as a Golgi marker, a 200-fold increase in activity was recorded after FFE enrichment relative to the initial developing xylem homogenate. In the final FFE fractions, an approximately 8-fold increase in specific GalS activity was recorded (Table 2) compared to the highest level of GalS activity previously reported for membrane preparations from compression wood [6]. GalS activity in the sCW sample was discovered to be lower at the 0.75/1.0 M interface compared to the 1.0/1.6 M interface, indicating that only a minority of Golgi membranes had moved up through the 1.0 M sucrose. This was not the case in the mCW sample and so is likely to be a result of compositional differences between severe and mild compression wood. Compression wood has an increased content of lignin [6]. Aglycones formed from coniferyl and sinapyl alcohols undergo radical coupling and so may lead to increased cross-linking and decreased separability in compression wood samples. If ferulates and diferulates are incorporated into pine lignin as in grasses [31], then this may promote polymer-lignin cross linking and could have further decreased separability of lignin-enriched compression wood samples. Phenolic compounds may act to cross link membrane proteins and limit separation of Golgi membranes, especially in severe compression wood samples. The cytoskeleton is well known to play an important role in cell wall development [32], so it is possible that as-yet unknown differences in cytoskeletal structure between mCW and sCW samples could have altered their behavior during electrophoresis. Specific GalS activity post-FFE would likely have been considerably higher had it been possible to further optimize sucrose density centrifugation techniques for pine xylem samples. In mCW, GalS activity was highest in the zone containing the most known Golgi-localized proteins, supporting previous suggestions that pine GalS was a Golgi localized enzyme based on its presence in microsomes [6].

GalS activity was detected in all pooled fractions of sCW which contained proteins (P1 to P4, Figure 2) but activity levels were markedly higher in P2 and P3, of which P3 contained the highest activity (Table S5 in File S1). In the sCW sample the highest GalS activity was observed in fraction sCW-P3 (Table 2). As may have been the case with the sucrose density gradient, compositional differences between sCW and mCW such as phenolic content, could have affected membrane separation. The pronounced presence of cytoskeletal components observed in this study and previously [13] may also have played a role. Cytoskeletal attachments may make it more difficult for Golgi vesicles to be separated from other cellular components, so only a sub-population of Golgi membranes might have been able to migrate further towards the anode during FFE. Interestingly, proteins homologous to actin depolymerizing factor, although relatively abundant in mCW, are absent from sCW (Table S4 in File S1). Actin was one of the most abundant proteins identified in both mCW and sCW (Tables S1 to S3 in File S1) so any alterations to the cytoskeleton between mCW and sCW could have impacted the capacity for electrophoretic separation of Golgi membranes from other cellular compartments. FFE appeared to partially separate Golgi membranes from most other sources of contamination (Table 4). However, Golgi purity levels were much lower than the ca. 80% purity achieved for Arabidopsis [18] where the presence of cytoskeletal proteins was minimal, indicating that the high load of cytoskeletal components may could also be affecting electrophoretic membrane separation. In future studies, steps should be included that maximize cytoskeletal disruption and removal of phenolic compounds such as polyvinylpyrrolidone [33] in the initial enrichment prior to FFE. It is possible that FFE could have a much greater impact on the separation of Golgi membranes from contaminating organelles in this case.

### Functional categorization of proteins in mCW and sCW samples

Categorizing proteins by function can reveal changes in tissue metabolism and their downstream effects in protein expression. This study identified an unprecedented number of proteins involved in cell wall biosynthesis of pine and so a comparison of functional categories between sCW and mCW samples could show metabolic changes associated with compression wood formation. However, very little change was observed in the proportional size of protein categories between sCW and mCW when functional categories were assigned. This prompted an analysis at the individual protein level, not only of differences between types of compression wood, but of all pine Golgi proteins involved in cell wall metabolism.

Compression wood typically has very high levels of β-1,4-galactan with a high degree of polymerization and lignin [4,34]. Xylan and glucomannan are present in both severe and medium compression wood and are thought to be necessary for the formation of lignin [5,34,35]. Members of the CSLA clade of GT2 have been shown to synthesize mannan [36] and the high number of proteins homologous to CSLAs in both sCW and mCW samples (Table 5) strongly suggests glucomannan synthesis is occurring. In addition to GT2, members of GT8 and GT47 were highly expressed in microarray data from poplar [37], implicating their significance in wood formation. Correspondingly, several members of GT47 are present in both sCW and mCW samples, including one homologue to GUT1. In Arabidopsis, GUT1 is critical to correct formation of glucuronoxylan [38], suggesting that glucuronoxylan may also be a key component of compression wood in pine. Although fewer proteins were identified in sCW samples, some proteins were found only in sCW and not mCW. Of these proteins, one was homologous to a GT8 protein. PARVUS, one of the GAUT-like (GATL) proteins in GT8 is involved in synthesis of the reducing end, and therefore initiation of synthesis, of xylan. A functional orthologue has been found in poplar [39]. The two other GT8s are most homologous to GAUT8 and GAUT9, which may be involved in pectin biosynthesis in Arabidopsis [40]. These GAUTs are common to sCW and mCW, making the GT8 like protein, unique to sCW and thus an interesting target for further study in compression wood formation. Cellulose synthase complex members (CESAs) were common in both sCW and mCW. Although compression wood has a lower cellulose content than tension wood [41], proteins homologous to cellulose synthase complex members (CESAs) were identified twice as frequently in sCW than mCW. Cellulose is made of β(1→4) linked D-glucose residues [42]. Analysis of the neutral sugars present in the mCW and sCW samples showed that sCW had slightly lower glucose content than mCW, despite the increase in CESA homologues (Table 1). Such contradiction within this study and with previous findings merits further investigation into cellulose content and the actual function of the CESA homologues in pine. The RGP was identified multiple times but only in mCW fractions. This points towards more arabinogalactan proteins in mCW, although previous studies have detected them in severe compression wood [4]. No candidates for β-1,4-galactan synthase GTs were identified, which is surprising considering the increase in GalS activity in fractions P2 and P3 and increased galactose content in sCW (Table 1). However, it is not known how this increase in activity would equate to protein abundance relative to those proteins most easily identified in the present study. Without a comprehensively annotated pine genome it is also possible the enzyme was identified here but wrongly assigned to a related protein category.

## Conclusion

In summary, FFE has proved to be a good method for increasing both the purity of Golgi preparations from developing xylem of pine and has proved to be an excellent tool in increasing the number of identified Golgi proteins from pine. There is clearly potential for additional improvements to this technique, through optimization of density centrifugation steps and the use of salt washes or depolymerizing factors to disrupt cytoskeletal attachments and enhance migration during electrophoresis. Nevertheless, this study has advanced our knowledge of some of the most abundant pine Golgi proteins involved in wood formation and, despite the qualitative nature of this work, has given an indication of proteins that could be important targets for further research into severe compression wood formation. In combination with forthcoming advances in sequencing and annotation of the pine genome, proteomic characterization of the Golgi apparatus will be a very useful platform for targeted cell wall engineering for improvements in wood quality and biofuel yield from timber-producing species.

## Supporting Information

File S1 **Figure S1, Tables S1-S6.** Figure S1. Image of the three-year-old pine tree (*Pinus* *radiata* D. Don) sourced from Swanton Pacific Ranch Davenport (CA, USA) that was used to harvest developing xylem for Golgi preparations. After sections were cut, logs were inspected to determine whether compression wood had formed. Logs were then placed on ice for transport back to the laboratory. Table S1. Proteins identified from fractions mCW-P2, mCW-P3, sCW-P2 and sCW-P3 after FFE enrichment of Golgi membranes from developing xylem of pine. Protein identifications were made with one or more significantly matching peptides by Mascot (p < 0.05) against Arabidopsis proteins (TAIR10). Column headers are standard outputs from the Mascot search engine (http://www.matrixscience.com/help/export_help.html). Table S2. Proteins identified from fractions mCW-P2, mCW-P3, sCW-P2 and sCW-P3 after FFE enrichment of Golgi membranes from developing xylem of pine. Protein identifications were made with one or more significantly matching peptides by Mascot (p < 0.05) against Viridiplantae proteins downloaded from GenBank (May 2012). Column headers are standard outputs from the Mascot search engine (http://www.matrixscience.com/help/export_help.html). Table S3. Proteins identified from fractions mCW-P2, mCW-P3, sCW-P2 and sCW-P3 after FFE enrichment of Golgi membranes from developing xylem of pine. Protein identifications were made with one or more significantly matching peptides by Mascot (p < 0.05) against *Pinus* spp. proteins at GenBank (July 2012). Column headers are standard outputs from the Mascot search engine (http://www.matrixscience.com/help/export_help.html). Table S4. Subcellular localization and functional matrix for fractions mCW-P2, mCW-P3, sCW-P2 and sCW-P3. Numbers indicate the protein hit number (prot_hit_num) from Table S1 (Arabidopsis), Table S2 (Viridiplantae) or Table S3 (pine). Subcellular locations were determined using the SUBcellular Arabidopsis (SUBA) database. Proteins were allocated a functional assignment using gene annotation information or BLAST analysis. Table S5. Galactan synthase (GalS) activity levels in FFE-purified fractions of developing xylem tissue from samples mCW and sCW. Table S6. Assignment of MapMan functional categories to proteins identified from mCW and sCW samples using the Arabidopsis database (TAIR10). Protein data were obtained from Table S1.
(ZIP)
