# Supplementary material for: Golgi Enrichment and Proteomic Analysis of Developing Pinus radiata Xylem by Free-Flow Electrophoresis
Source: PLoS One. 2013 Dec 26;8(12):e84669. doi: 10.1371/journal.pone.0084669 (PMC3887118; doi:10.1371/journal.pone.0084669)
Supplement: File S1 — Figure S1, Tables S1-S6. Figure S1. Image of the three-year-old pine tree (Pinus radiata D. Don) sourced from Swanton Pacific Ranch Davenport (CA, USA) that was used to harvest developing xylem for Golgi preparations. After sections were cut, logs were inspected to determine whether compression wood had formed. Logs were then placed on ice for transport back to the laboratory. Table S1. Proteins identified from fractions mCW-P2, mCW-P3, sCW-P2 and sCW-P3 after FFE enrichment of Golgi membranes from developing xylem of pine. Protein identifications were made with one or more significantly matching peptides by Mascot (p < 0.05) against Arabidopsis proteins (TAIR10). Column headers are standard outputs from the Mascot search engine (http://www.matrixscience.com/help/export_help.html). Table S2. Proteins identified from fractions mCW-P2, mCW-P3, sCW-P2 and sCW-P3 after FFE enrichment of Golgi membranes from developing xylem of pine. Protein identifications were made with one or more significantly matching peptides by Mascot (p < 0.05) against Viridiplantae proteins downloaded from GenBank (May 2012). Column headers are standard outputs from the Mascot search engine (http://www.matrixscience.com/help/export_help.html). Table S3. Proteins identified from fractions mCW-P2, mCW-P3, sCW-P2 and sCW-P3 after FFE enrichment of Golgi membranes from developing xylem of pine. Protein identifications were made with one or more significantly matching peptides by Mascot (p < 0.05) against Pinus spp. proteins at GenBank (July 2012). Column headers are standard outputs from the Mascot search engine (http://www.matrixscience.com/help/export_help.html). Table S4. Subcellular localization and functional matrix for fractions mCW-P2, mCW-P3, sCW-P2 and sCW-P3. Numbers indicate the protein hit number (prot_hit_num) from Table S1 (Arabidopsis), Table S2 (Viridiplantae) or Table S3 (pine). Subcellular locations were determined using the SUBcellular Arabidopsis (SUBA) database. Proteins were al [file pone.0084669.s001.zip › TableS5.pdf]

Table S5. Galactan synthase (GalS) activity levels in FFE-purified fractions of developing xylem tissue from samples mCW and sCW.

**Sample**	**Specific activity [pmol min^{-1} mg^{-1} protein]**	**Total activity [pmol min^{-1}]**
mCW-P1	756.3	6.0
mCW-P2	7304.9	29.2
mCW-P3	753.9	67.8
mCW-P4	ND	ND
mCW-P5	0.0	0.0
sCW-P1	3131.8	37.6
sCW-P2	12318.8	197.1
sCW-P3	34662.8	588.0
sCW-P4	4320.1	45.3
sCW-P5	0.0	0.0

ND: not determined since an FFE ‘peak 4’ from mCW was not present, therefore samples in that range were not collected.
